# CXCL12/CXCR4 Axis Activation Mediates Prostate Myofibroblast Phenoconversion through Non-Canonical EGFR/MEK/ERK Signaling

**DOI:** 10.1371/journal.pone.0159490

**Published:** 2016-07-19

**Authors:** José A. Rodríguez-Nieves, Susan C. Patalano, Diego Almanza, Mehrnaz Gharaee-Kermani, Jill A. Macoska

**Affiliations:** Center for Personalized Cancer Therapy and Department of Biology, University of Massachusetts, Boston, Massachusetts; II Università di Napoli, ITALY

## Abstract

Benign prostate hyperplasia (BPH), an enlargement of the prostate common in aging in men, is associated with urinary voiding dysfunction manifest as Lower Urinary Tract Symptoms (LUTS). Although inflammation and abnormal smooth muscle contractions are known to play key roles in the development of LUTS, tissue fibrosis may also be an important and previously unrecognized contributing factor. Tissue fibrosis arises from the unregulated differentiation of fibroblasts or other precursor cell types into myofibroblasts, which is usually accomplished by activation of the TGFβ/TGFβR axis. Previously we reported that the CXC-type chemokines, CXCL5, CXCL8 and CXCL12, which are up-regulated in the aging in the prostate, can drive this differentiation process as well in the absence of TGFβ. Based on this data we sought to elucidate the molecular mechanisms employed by CXCL12, and its receptor CXCR4, during prostate myofibroblast phenoconversion. The results of these studies suggest that CXCL12/CXCR4-mediated signaling events in prostate myofibroblast phenoconversion may proceed through non-canonical pathways that do not depend on TGFβ/TGFβR axis activation or Smad signaling. Here we report that CXCL12/CXCR4 axis activation promotes signaling through the EGFR and downstream MEK/ERK and PI3K/Akt pathways during myofibroblast phenoconversion, but not through TGFβ/TGFβR and downstream Smad signaling, in prostate fibroblasts undergoing myofibroblast phenoconversion. We document that EGFR transactivation is required for CXCL12-mediated signaling and expression of genes associate with myofibroblast phenoconversion (α-SMA, COL1a1). Our study successfully identified TGFβ/TGFβR-independent molecular mechanisms that promote CXCL12/CXCR4-induced myofibroblast phenoconversion. This information may be crucial for the development of novel therapies and potential biomarkers for prostatic fibrosis.

## Introduction

Benign prostate hyperplasia (BPH) is an aging related prostatic enlargement that affects the majority of older men[1]. Complications can arise from BPH that affect the quality of life of patients. Some of this complications, or symptoms, include nocturia, incomplete voiding of the bladder, weak stream, and are collective known as lower urinary tract symptoms (LUTS) [1–3]. LUTS is treated with a wide range of pharmaceutical agents that target two aspects of LUTS: hyperplasia and smooth muscle dysfunction[4,5]. 5-alpha-reductase inhibitors, which treat hyperplasia, target the androgen receptor to reduce the size of the prostate by inhibiting cell proliferation and therefore decreasing pressure in the segment of the urethra that goes through the prostate. Alpha-1-adrenergic receptor antagonists target smooth muscle function to relax the muscular tissue surrounding the urethra, allowing better urine flow in these patients[6,7]. However, these therapeutic approaches are not always effective, suggesting that additional pathobiologies may contribute to LUTS in aging men[5,8].

Recent data from several groups, including our own, has shown that inflammation plays a role in the development of collagen deposition and consequent tissue remodeling associated with fibrosis in multiple organs, including the prostate[9–13]. Fibrosis is the result of unregulated tissue repair[14,15]. Tissue repair involves several stages that include inflammation, the recruitment immune, vascular, and stromal cell types, extracellular matrix deposition and tissue remodeling. As part of this process, resident fibroblasts and other progenitor cells respond to inflammatory signals through proliferation and phenoconversion to a myofibroblast phenotype[11]. These newly formed myofibroblasts, in turn, deposit the extracellular matrix that promotes wound closure. Upon wound repair, myofibroblasts either migrate out of the wound area or undergo apoptosis[16]. If myofibroblasts abnormally persist, excess ECM is deposited, resulting in scarring and tissue stiffening[17].

Inflammatory infiltrate is commonly observed in the prostate during aging, and is especially notable in enlarged glands[9,18]. In addition, our group has previously shown that pro-inflammatory proteins are secreted by aging prostate fibroblasts, particularly CXC-type chemokines[19,20]. We have shown that CXCL5, CXCL8 and CXCL12 can promote the phenoconversion of prostate fibroblasts to myofibroblasts[21]. However, the molecular mechanisms underlying these CXC-type chemokine-mediated phenoconversion events were not known. In this manuscript, we examined whether CXCL12/CXCR4-mediated myofibroblast phenoconversion was coupled to canonical TGFβ/TGFβR signaling. The results of these studies demonstrate that CXCL12/CXCR4-mediated myofibroblast phenoconversion is accomplished through non-canonical MEK/ERK signaling pathways. This finding is significant because it shows that multiple signaling pathways may require targeting in order to develop effective anti-fibrotic agents for use in the lower urinary tract.

## Experimental Precedures

### Cell culture and treatments

N1 prostate fibroblasts[22] and primary prostate fibroblasts[23] were grown in 5% HIE culture media (Ham’s F-12, 5% FBS, Insulin [5 μg/mL], EGF [10 ng/mL], Hydrocortisone [1 μg/mL], Fungizone [0.5 μg/mL], Gentamicin [0.05 mg/mL]). Prior to treatment, cells were serum starved overnight using SF HIE (Ham’s F12, EGF [50 ng/mL], 0.1% BSA, Insulin [5 μg/mL], Transferrin [5 μg/mL], 50 μM sodium selenite, 10 uM 3,3’, 5-triiodo-L-thyronine, Hydrocortisone [1 μg/mL], Fungizone [0.5 μg/mL], Gentamicin [0.05 mg/mL]) Fibroblasts were then treated with 100pM of human CXCL12 (R&D Systems) or 50 ng/ml EGF, or 0.01% BSA vehicle, and/or with 4 ng/mL TGFβ (R&D Systems) or 20nM citrate vehicle, and collected at the desired time points. For inhibitor treatments, fibroblasts were treated with chemical inhibitors 2 hours prior to CXCL12 treatment. The chemical inhibitors used were the following: AMD3100 (25 μM, Sigma-Aldrich), SB431542 (500 nM, Sigma-Aldrich), AG1478 (500 nM, Invitrogen).

### Western Blotting

Following the desired treatment, cells were collected in protease inhibitor cocktail (PIC)-containing PBS. Cells were lysed in Radioimmunoprecipitation assay (RIPA) Buffer (10 mM Tris-Cl (pH 8.0), 1 mM EDTA, 0.5 mM EGTA, 1% Triton X-100, 0.1% sodium deoxycholate, 0.1% SDS, 140 mM NaCl). Protein quantification was carried out using Bio-Rad OneStep Bradford reagent and an Elx800 Microplate Reader (Bio-Tek) with Gen5 software. Protein lysates were prepared for electrophoresis using 4X Lithium Dodecyl Sulfate (LDS) Sample Buffer and β-ME and run in 8% Tris-Gly SDS precast gels (Life Technologies), then transferred to nitrocellulose membranes (Thermo Scientific) using the Pierce G2 blotter. Membranes were blocked using a 5% Milk TBS-T solution for one hour. Primary antibody incubation was performed using a 5% BSA TBS-T (50 mM Tris, 150 mM NaCl, 0.05% Tween 20, pH 7.6) solution for EGFR (#2232), Y1068 pEGFR (#2234), Akt (#9272), S473 pAkt (#9271), Smad3 (#9523), S423/425 pSmad3 (#9520), ERK 1/2 (#9102), T202/Y204 pErk (#9101), Y416 pSrc (#2101), Src (#2108), TGFβRI (#3712), GAPDH (#2118) from Cell Signaling Technologies, actin (#SC1615) from Santa Cruz Biotechnology, and α-smooth muscle actin (#ab5694) CXCR4 (#ab2074), collagen 1 (AbCam #ab34710), from AbCam antibodies. Secondary antibody incubations using Horse Radish Peroxidase, HRP, Conjugated anti-rabbit [1:5,000 dilution, Cell Signaling] or fluorescent anti-mouse/rabbit [1:50,000 dilution, LiCor Systems]) were performed for 1 hour at room temperature. Membranes were washed twice with TBS-T and scanned using the Odyssey CLx and c-Digit (LI-COR), for the detection of fluorescent and HRP-conjugated antibodies respectively. Immunoblots were quantified and analyzed using the ImageStudio software suite (LI-COR). Conditioned media westerns were conducted using media collected from treated cells. 4mLs of media were spun for 40 minutes at 4,000rpm at 4C using Amicon Ultracel 3K (Millipore #UFC800324) to concentrate the samples to 250uL. Concentrated media was loaded in equal volumes (15uL) for SDS-PAGE and western blot analysis. Collagen 1 (AbCam #ab5694) immunoblot images were normalized to Ponceau S (0.1% Ponceau S in 5.0% acetic acid, Sigma #P7170) staining using ImageStudio software (LiCor). Normalized images were plotted as total value over Ponceau S staining to compensate for loading differences.

### RNA Extraction and Gene Expression Analysis

N1 and primary prostate fibroblast cells were treated as above and subjected to RNA extraction using Trizol reagent (Invitrogen, Carlsbad, CA). Purified RNA was treated with DNase and qRT-PCR analysis was performed using a QuantStudio 12K Flex Real-Time PCR System, reagents and software (Applied Biosystems, Carlsbad, CA). For all experiments, 1 μg of RNA was reverse transcribed using a High Capacity cDNA Reverse Transcription Kit (Applied Biosystems, Carlsbad, CA). qPCR was performed using Assays on Demand (Applied Biosystems, Foster City, CA) according to the manufacturer’s instructions. Reactions were performed in triplicate, including no template controls and amplification of an endogenous control transcript, Larger Ribosomal Protein (RPLPO) to assess template concentration and loading precision. Results were analyzed for integrated and precision using the QuantStudio 12K Flex Software. Cycle numbers to threshold were calculated by subtracting averaged control from averaged experimental values and collagen 1α1 (COL1α1), α-smooth muscle actin (ACTA2) and TGFβ (TGFβ1) transcript levels were normalized to those of RPLPO using the Pfaffl method. Gene-specific assays were Hs0016400_m1 for COL1α1, Hs00909449_m1 for ACTA2, Hs00998130 for_m1 for TGFβ1, and Hs99999902_m1 for RPLPO (Applied Biosystems, Carlsbad, CA).

### Immunofluorescence

N1 prostate fibroblasts were plated on chamber slides coated with 10ug/ml fibronectin (Sigma-Aldrich, St. Louis, MO). Cells were washed with phosphate buffered saline (PBS), and then switched to SF HIE media for 24 hr. The cells were then treated with 0.1% BSA in PBS (vehicle) or 100pM CXCL12 for 48 hours at 37°C in a 5% CO2 incubator in the absence or presence of small molecule inhibitors or antibodies as described above. Primary antibodies were diluted in blocking solution and included 1:200 dilution FITC-conjugated mouse monoclonal anti-α-smooth muscle actin (α SMA), and 1:100 dilution biotin-conjugated rabbit polyclonal anti-collagen type 1 (COL1) (Rockland Immunochemicals, Gilbertsville, PA). Cells were counterstained for 5 min with 1 mg/ml DAPI (Molecular Probes, Eugene, OR) in Tris-Buffered Saline/Tween 20, washed three times for 5 min each with TBST, and mounted in an Aqua-mount (Lerner Laboratories, PA). Photomicrographs were taken on an Olympus BX51 fluorescence microscope. PE-Cy 5 streptavidin (BD Pharmingen San Diego, CA) or anti-mouse Alexa 488 or Alexa 555 (Invitrogen, Carlsbad, CA) secondary antibodies were used at 1:2000 dilutions. Control mouse IgG2a (Sigma-Aldrich, St. Louis, MO) and rabbit IgG biotin conjugate (Rockland Immunochemicals, Gilbertsville, PA) were used at 1:2000 dilution. Fluorescent images at 40x were digitally captured using an Olympus BX51 photomicroscope with mercury bulb and Olympic filter cubes U-MU (dichroic mirror DM400, excitation filter BP330–385, barrier filter BA420), U- MWB (dichroic mirror DM500, excitation filter BP450–480, barrier filter BA515) and U-MSWB (dichroic mirror DM570, excitation filter BP510–550, barrier filter BA590).

### siRNA Transfection

siRNA against CXCR4 (ON-TARGET plus SMARTpool Human CXCR4 # L-005139-00) and TGFβRI (ON-TARGET plus SMARTpool Human TGFβRI # L-003929-00) and scramble control siRNA (#SR30004) were purchased from Dharmacon (Lafayette, CO), and Origene (Rockville, MD) respectively. N1 cells were seeded at 50% density the day before transfection in six-well plates, and 20 nM siRNA or scramble transfections were performed with DharmaFECT 1 transfection reagent from Dharmacon according to manufacturer’s instructions, using a dilution ratio of 1:500. Cells were then switched to serum-free media for 18 h, followed by treatment with vehicle 100 pM CXCL12 or 4 ng/mL TGF-β for 1h for signaling analysis, and 24 hours for gene expression analysis. Western blot analysis of signaling event and RNA isolation and quantitative real time PCR analysis were performed as described above.

### Statistical Analysis

Averages and standard deviations were calculated and compared using Student’s t-tests. In all tests, p < .05 was considered statistically significant.

## Results

### CXCL12/CXCR4 axis-mediated myofibroblast phenoconversion is not coupled to Smad3 phosphorylation

**Fig 1:**
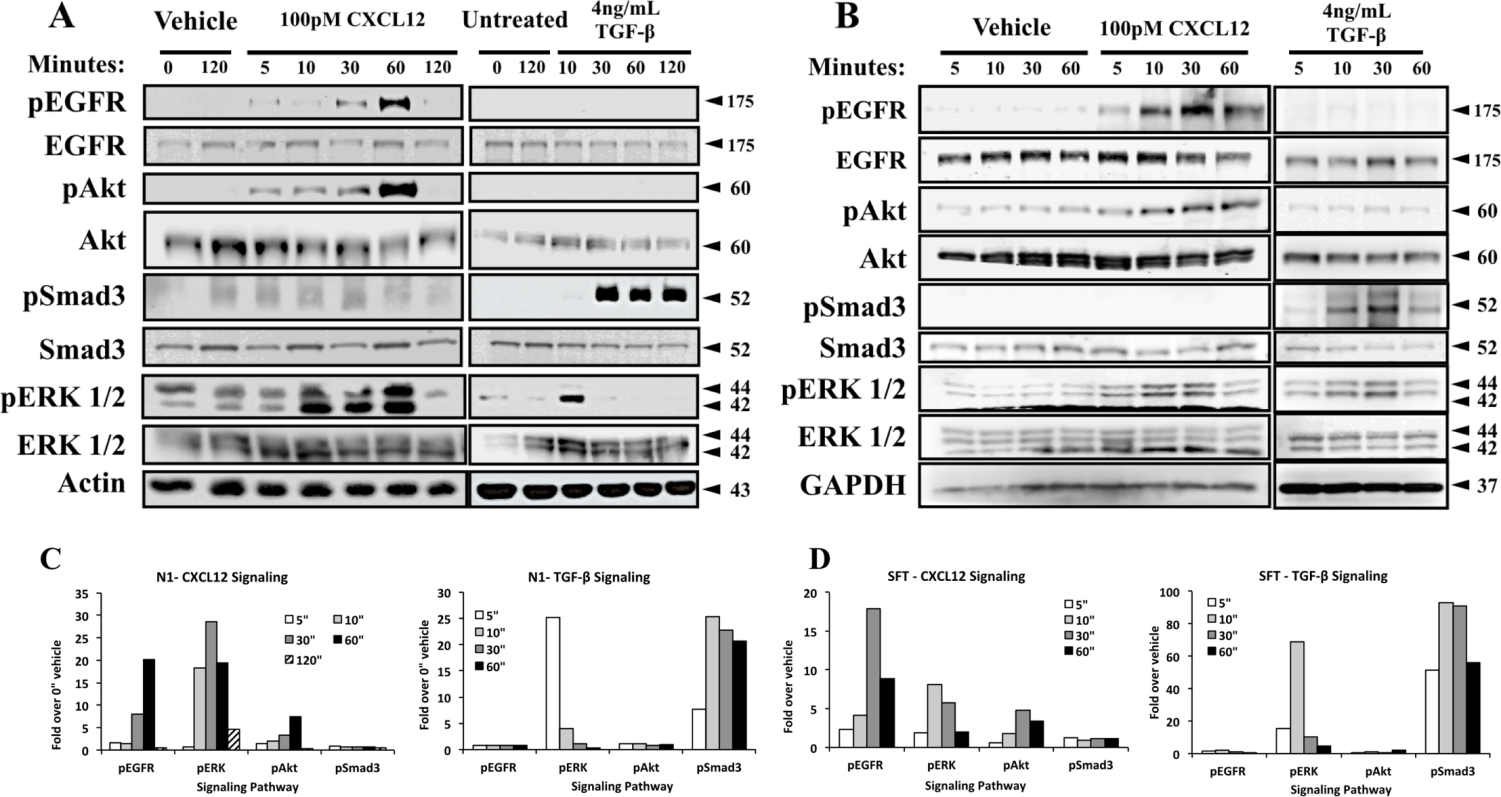
CXCL12/CXCR4 axis-mediated myofibroblast phenoconversion is not coupled to Smad3 phosphorylation. N1 fibroblasts (**A**) and primary prostate fibroblasts (**B**) were treated in defined serum-free Ham’s media with CXCL12 (100pM), or 0.01% BSA vehicle, and TGFβ (4 ng/mL) or 20mM citric acid vehicle. In both types of cells EGFR, Akt and Erk1/2 were phosphorylated upon CXCL12 treatment. TGFβ treatment activated Smad-mediated signaling and transient Erk1/2 phosphorylation, but not EGFR or Akt phosphorylation. Total antibodies for each kinase, as well as GAPDH and actin, were used as loading control. Protein molecular weight in kilodaltons is indicted by arrows. Signal intensity quantification (**C**) for N1 fibroblasts shows that EGFR activation occurs at 5 minutes post-treatment, followed by a robust activation of Erk at 10 minutes as well as mild Akt activation. The same pattern was observed in primary fibroblasts (**D**), however EGFR activation surpassed that of Erk.

We have previously shown that both CXCL12 and TGFβ mediate the myofibroblast phenoconversion of prostate fibroblasts[21]. Many studies have shown that activation of the TGFβ/TGFβR axis promotes immediate Smad3 phosphorylation during myofibroblast phenoconversion[14,24–26]. Therefore, we first examined whether activation of the CXCL12/CXCR4 axis also promoted Smad3 phosphorylation during myofibroblast phenoconversion. To test this, N1 immortalized[22] or SFT1[23] primary prostate fibroblasts were treated in SF HIE media with either 100pM CXCL12 or 4ng/ml TGFβ. The results of these studies showed that activation of the CXCL12/CXCR4 axis rapidly but phosphorylated the EGFR at tyrosine 1068 as well as downstream MAPK/Erk and PI3K/Akt signaling pathways, but did not promote Smad3 phosphorylation, in both N1 (Fig 1A) and SFT1 (Fig 1B) cells. In contrast, TGFβ treatment strongly induced Smad3 phosphorylation as expected, but not that of EGFR or Erk in both cell cultures (Fig 1A and 1B). Signal intensity quantification (Fig 1C) showed that CXCL12 treatment induce EGFR activation by 5 minutes post-treatment, followed by robust activation of Erk and mild activation of Akt in N1 fibroblast. It is important to note that TGFβ treatment also activated Erk signaling at 5 minutes post-treatment, but not EGFR as observe in CXCL12 treatment of N1 fibroblasts. The same trend was observed in SFT fibroblasts (Fig 1D), although EGFR activation was stronger than Erk after CXCl12 treatment. Taken together, these data indicate that the CXCL12/CXCR4 axis promotes signaling through Smad-independent, non-canonical EGFR and downstream MAPK/Erk and PI3K/Akt pathways in the absence of TGFβ in both N1 and primary prostate fibroblasts. This clearly indicates that CXCL12 and TGFβ stimulate different intracellular signaling mechanisms to promote myofibroblast phenoconversion.

### CXCL12/CXCR4-mediated signaling and myofibroblast phenoconversion requires EGFR transactivation

**Fig 2:**
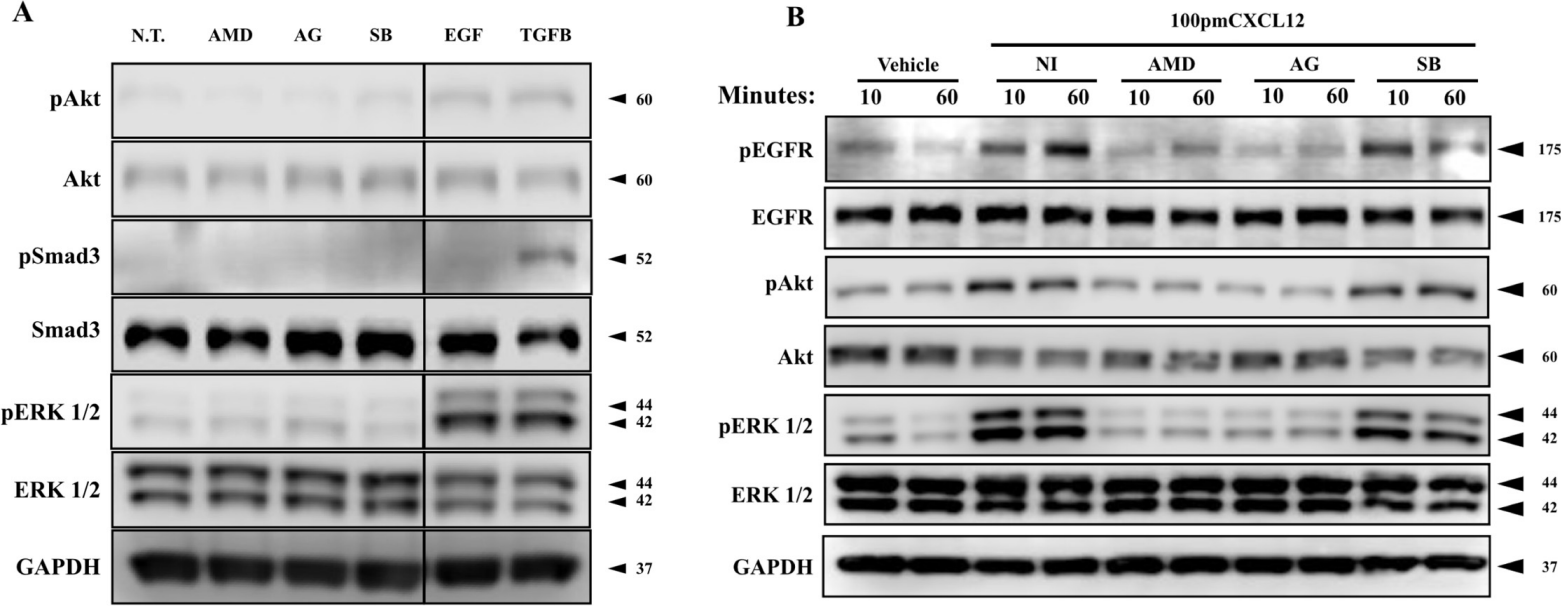
CXCL12/CXCR4-mediated signaling and myofibroblast phenoconversion requires EGFR transactivation. (**A**) N1 fibroblasts were pre-treated with chemical inhibitors of CXCR4 (25 μM AMD3100), EGFR (500 nM AG1478), and TGFβRI (500 nM SB431542) or with 50 ng/ml EGF or 4ng/ml TGFβ for three hours. No activation of downstream signaling kinases (Akt, smad3, Erk) was observed in inhibitor-only treatment of fibroblasts. (**B**) N1 fibroblasts were pre-treated with chemical inhibitors of CXCR4 (25 μM AMD3100), EGFR (500 nM AG1478), and TGFβRI (500 nM SB431542) for two hours prior to CXCL12 (100 pM) treatment. Treatment with AMD3100 and AG1478 completely ablated the phosphorylation and activation of downstream targets. Inhibition of TGFβRI activation, with SB431542, had no effect of CXCL12 signaling.

Previously our group had shown that intracellular signaling downstream of CXCL12/CXCR4 axis activation requires transactivation of the EGFR in prostate epithelial cells[27]. In order to determine whether EGFR transactivation is required for CXCL12/CXCR4-mediated signaling in prostate fibroblasts, N1 cells were pre-treated with inhibitors that block CXCR4 activation (25 μM AMD3100, AMD); EGFR auto-phosphorylation (500 nM AG1478, AG), or TGFβRI activation (SB431542, SB). Untreated cells demonstrate little or no basal phosphorylation of downstream signaling pathways SAkt, Smad3, or ERK (Fig 2A). When treated with EGF, N1 cells demonstrate low Akt and Smad3 phosphorylation but robust ERK phosphorylation (Fig 2A). In contrast, N1 cells treated with TGFβ demonstrated robust Smad3 phosphorylation but no Akt or ERK phosphorylation (Fig 2A). When treated with 100pM CXCL12, N1 cells evinced elevated levels of EGFR and ERK phosphorylation (Fig 1B). However, EGFR or downstream signaling pathways activation was not evident in cells pre-treated with AMD3100 or AG1478 prior to CXCL12 treatment (Fig 2B).These data suggest that activation of the CXCL12/CXCR4 axis promotes EGFR transactivation, and that this transactivation is necessary for downstream intracellular signaling in prostate fibroblasts. In contrast, N1 cells pre-treated with SB431542, small molecule inhibitor of the TGFβRI, showed uninterrupted CXCL12/CXCR4 axis signaling, suggesting that inhibition of the TGFβ/TGFβRI was not coupled to CXCL12/CXCR4 axis signaling.

**Fig 3:**
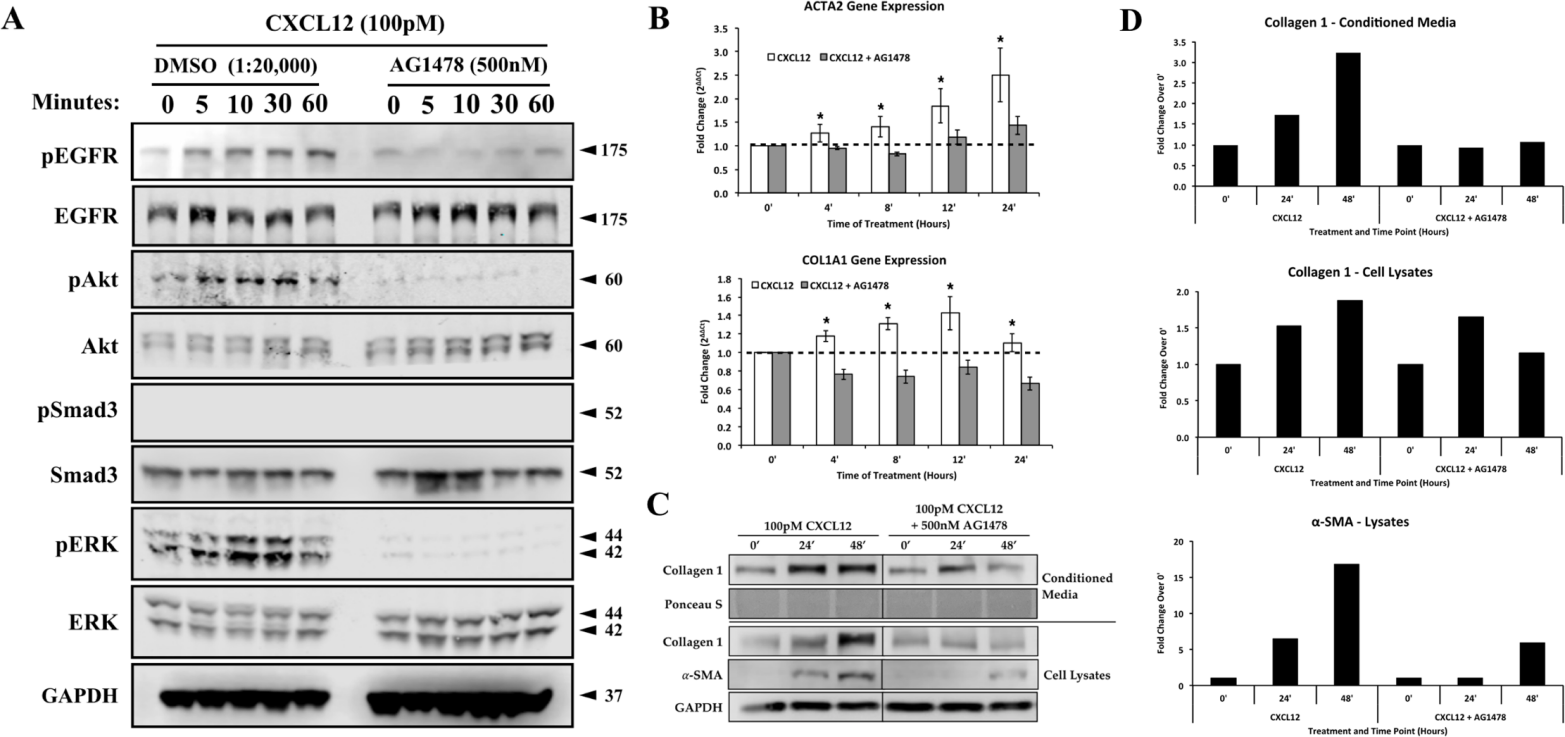
Activation of the CXCL12/CXCR4 and TGFβ/TGFβR axes independently promote ACTA2 and COL1A1 gene expression. (**A**) N1 fibroblasts were treated with vehicle (DMSO) or EGFR inhibitor (AG1478, 500 nM) prior to CXCL12 treatment. Phosphorylation of EGFR, Akt, Smad and ERK1/2 were assessed via western blot. (**B**) qRT-PCR analysis of myofibroblast marker expression after CXCL12 (100pM) treatment in the presence or absence of AG1478 (500 nM). Expression levels of α-smooth muscle actin (ACTA2) and collagen 1α1 (COL1α1) were analyzed over the course of 24 hours of treatment. Treatment with AG1478 reduced or ablated the CXCL12/CXCR4 -mediated stimulation of both the ACTA2 and COL1α1 genes. (**C**) Western blot analysis of fibroblast markers, α-smooth muscle actin and collagen 1, at 24 and 48 hours after CXCL12 treatment. Secretion and incorporation of collagen 1 was inhibited in the presence of AG1478, α-smooth muscle actin production was inhibited as well. (**D**) Signal intensity quantification for 7 and collagen 1 western blots. * = p-value < 0.05. Error bars, SE.

Based on these results we next examined the potential functional effects of EGFR inhibition on CXCL12/CXCR4-mediated transcription of genes that encode smooth muscle actin (ACTA2) or collagen 1 (COL1α1), both of which are transcribed during myofibroblast phenoconversion[21]. Pre-treatment with AG1478 prior to CXCL12 treatment completely inhibited CXCL12/CXCR4 axis-mediated intracellular signaling activation as observed by the lack of EGFR, ERK, or Akt phosphorylation (Fig 3A). As observed previously, activation of the CXCL12/CXCR4 axis did not promote Smad3 phosphorylation (Fig 3A). Quantitative RT-PCR studies showed that activation of the CXCL12/CXCR4 axis promoted transcription of both ACTA2 and COL1α1 at levels significantly higher than vehicle-cells (Fig 3B). Pre-treatment with the EGFR inhibitor, AG1478 ablated CXCL12/CXCR4 axis-induced transcription of both genes (Fig 3B). Western blot analysis of myofibroblast markers (Fig 3C) showed that pre-treatment with AG1478 inhibited the ability of fibroblast to express and secrete collagen 1, as well as to express α-smooth muscle actin when cells were treated with CXCL12. Western blot signal quantification (Fig 2D) showed that CXCL12-mediated extracellular collagen and α-smooth muscle actin were completely inhibited in the presence of AG1478. These results indicate that EGFR activation is a required step for CXCL12/CXCR4-mediated downstream signaling and myofibroblast marker expression

### The CXCL12/CXCR4 and TGFβ/TGFβRI axes function independently to promote myofibroblast phenoconversion

**Fig 4:**
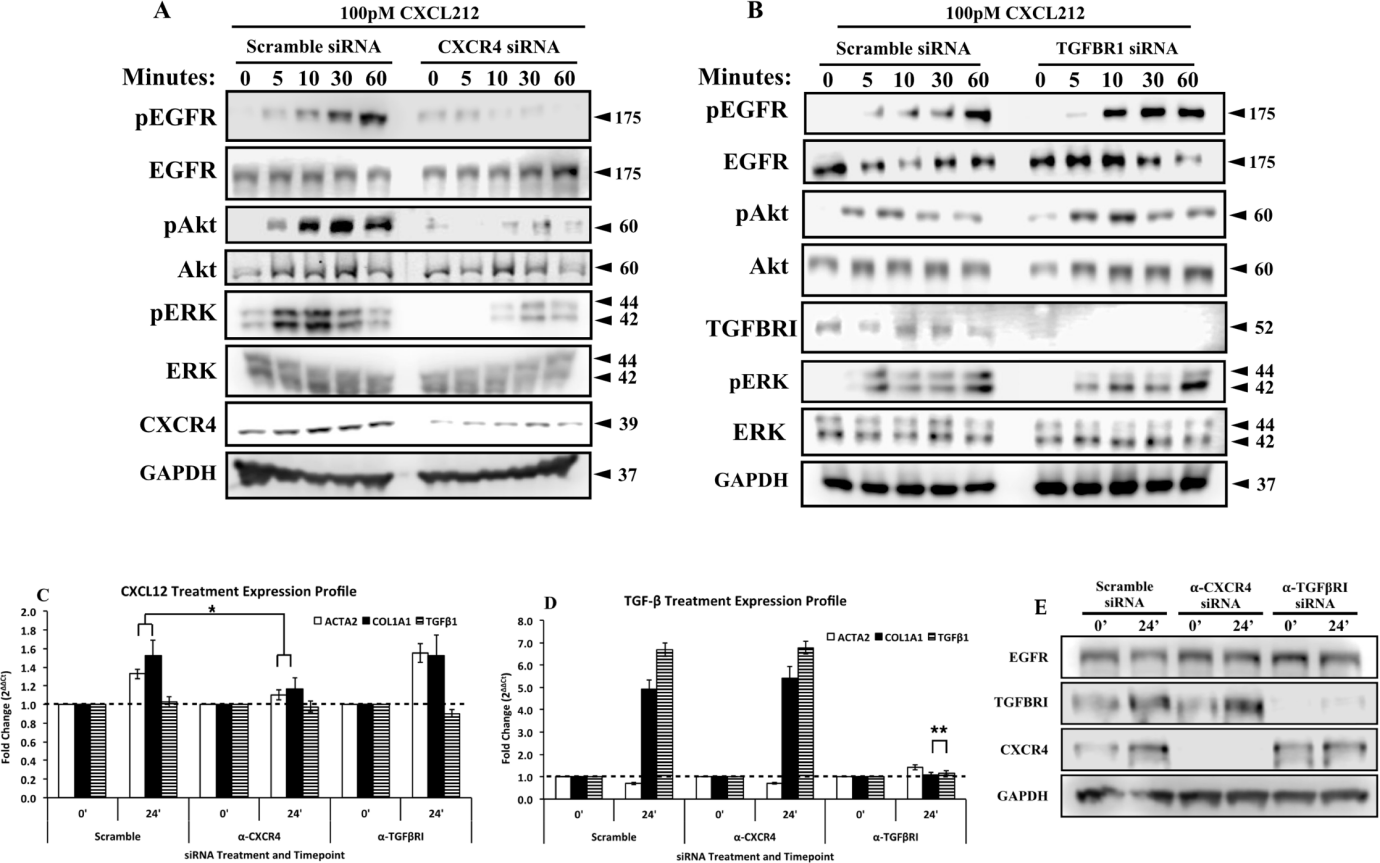
The CXCL12/CXCR4 and TGFβ/TGFβRI axes function independently to promote myofibroblast phenoconversion. N1 fibroblasts were transfected with 20nM Scramble, anti-CXCR4 (**A**), and anti-TGFβRI (**B**) siRNAs for 24 hours and treated with CXCL12 (100pM) for 1 hour. CXCR4 partial knockdown (**A**) reduced the CXCL12-mediated activation of EGFR, Akt and Erk1/2. However, TGFβRI knockdown (**B**) had no effect in the CXCL12-mediated activation of EGFR, Akt and ERK1/2. siRNA-mediated knockdown were validated using antibodies against target receptor. Total antibodies for each kinase and actin were used as loading control (Western blot quantification is provided in S1 Fig). N1 fibroblasts were transfected with 20nM Scramble, anti-CXCR4, and anti-TGFβRI siRNAs for 24 hours and treated with CXCL12 (100 pM) (**C**) and TGFβ (4 ng/mL) (**D**) for 24 hours. qRT-PCR analysis of α-smooth muscle actin (ACTA2), collagen 1α1 (COL1α1) and TGFβ (TGFβ 1) was performed. (**E**) Western blot analysis to confirm effective knockdown of CXCR4 and TGFβRI during gene expression analysis. * = p-Value <0.05, ** = p-Value < 0.001. Error bars, SE.

**Fig 5:**
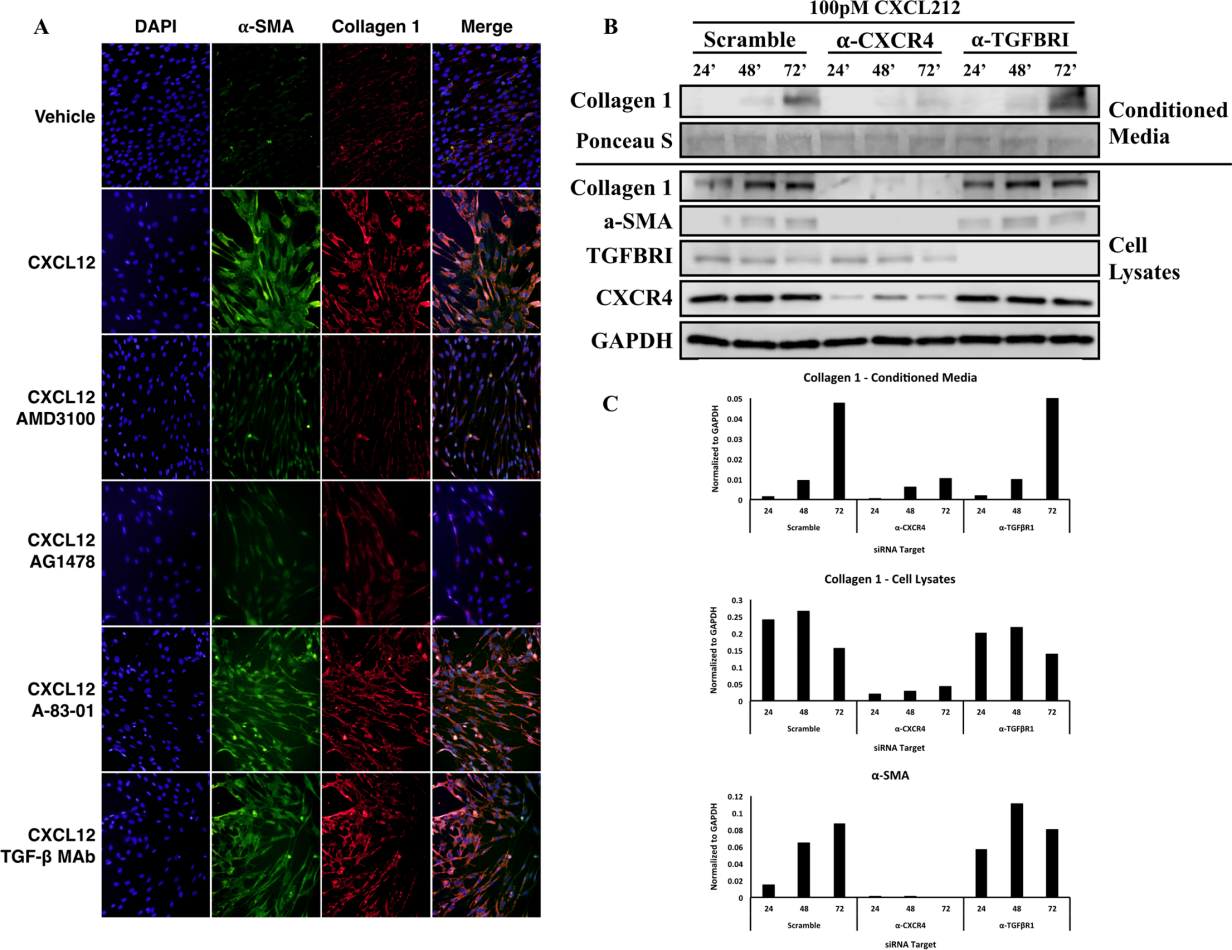
Inhibition TGFβ and TGFβRII-mediated signaling does not affect CXCL12-mediated myofibroblast phenoconversion. (**A**) Immunofluorescence analysis of N1 fibroblasts left untreated (-CXCL12) or treated with 100pM CXCL12 (CXCL12) for 48 hours in the absence or presence of CXCR4 (250 μM AMD3100), EGFR (250 uM AG1478), ALK-5 (TGFβRII) (20μM A-83-01) small molecular inhibitors or an antibody against TGFβRII (200 ng/ml TGFβ MAb). Figure depicts photomicrographs images of cells stained for α-smooth muscle actin (green) or collagen 1 (red) proteins or DAPI (blue nuclear stain); orange color indicates α-smooth muscle actin and collagen 1 colocalization in the merged image. (**B**) Western blot analysis of fibroblast markers, α-smooth muscle actin and collagen 1 at 24, 48 and 72 hours after CXCL12 (100pM) of scramble, anti-CXCR4, and anti-TGFβRI siRNAs transfected fibroblasts. CXCL12-driven expression of myofibroblasts markers does not require the presence or activation of TGFβRI. (**C**) Quantification of western blot images for myofibroblast markers.

In order to determine whether the CXCL12/CXCR4 and TGFβ/TGFβRI axes functioned together, in parallel, or independently to promote myofibroblast phenoconversion, we performed siRNA-mediated knockdowns of TGFβRI and CXCR4 to determine whether CXCR4 alone or both CXCR4 and TGFβRI were required for CXCL12-mediated myofibroblast phenoconversion. N1 cells knocked down for the CXCR4 receptor then treated with CXCL12 demonstrated greatly reduced EGFR, Akt and ERK1/2 phosphorylation when compared to control scramble siRNA transfected cells (Fig 4A). However, N1 cells knocked down for the TGFβRI receptor demonstrated robust phosphorylation of EGFR, Akt and ERK1/2 compared to control scramble siRNA transfected cells (Fig 4B). These results indicate that CXCL12-mediated intracellular signaling is coupled only to CXCR4, and does not require TGFβRI. To further test this finding, N1 cells knocked down for CXCR4 or TGFβRI were treated with CXCL12 and monitored for ACTA2 and COL1α1 gene expression. As shown in Fig 5C, CXCR4 knock-down, but not TGFβRI knock-down cells, demonstrated significantly reduced ACTA2 and COL1α1 transcript levels compared to control scramble siRNA transfected cells in response to treatment with CXCL12. Conversely, cells knocked down for TGFβRI, but not CXCR4, demonstrated significantly reduced ACTA2 and COL1α1 transcript levels compared to control scramble siRNA transfected cells in response to treatment with TGFβ (Fig 4D and 4E).

Based on these findings, immunofluorescence studies were performed to examine potential axes cross talk at the protein level. As shown in Fig 5A, N1 cells treated with CXCL12 demonstrate co-expression of alpha smooth muscle actin (αSMA) and collagen 1 (COL1) proteins and the acquisition of a stellate morphology consistent with myofibroblast phenoconversion. Co-expression was ablated in cells pre-treated with the CXCR4 inhibitor AMD3100, and the EGFR inhibitor AG1478 (Fig 5A). In contrast, cells pre-treated with either the TGFβR1 small molecule inhibitor A-83-01 or an anti-TGFβR1 monoclonal antibody then treated with CXCL12 demonstrated robust αSMA and COL1a1 co-expression and myofibroblast morphology (Fig 5A). In addition to analyzing myofibroblast phenoconversion, siRNA-mediated knockdowns of CXCR4 and TGFβR1 were conducted in N1 fibroblast to address the role of TGFβR1 and TGFβR1-mediated signaling in myofibroblast marker expression after CXCL12 treatment. As shown in Fig 5B, CXCL12-driven expression of myofibroblast markers do not require the presence TGFβR1. TGFβR1 siRNA-mediated knockdown did not exhibit any effects on the expression of α-smooth muscle actin and collagen 1 when treated with CXCL12. However, siRNA-mediated knockdown of CXCR4 completely ablated the expression of α-smooth muscle actin and collagen 1.

Taken together, the siRNA and immunofluorescence data demonstrate that the CXCL12/CXCR4 axis promotes the transcription and expression of genes and proteins associated with myofibroblast phenoconversion in the absence of TGFβ or the TGFβ/TGFβR signaling axis. This suggests a novel and non-canonical mechanism by which a pro-inflammatory chemokine promotes myofibroblast phenoconversion.

**Fig 6:**
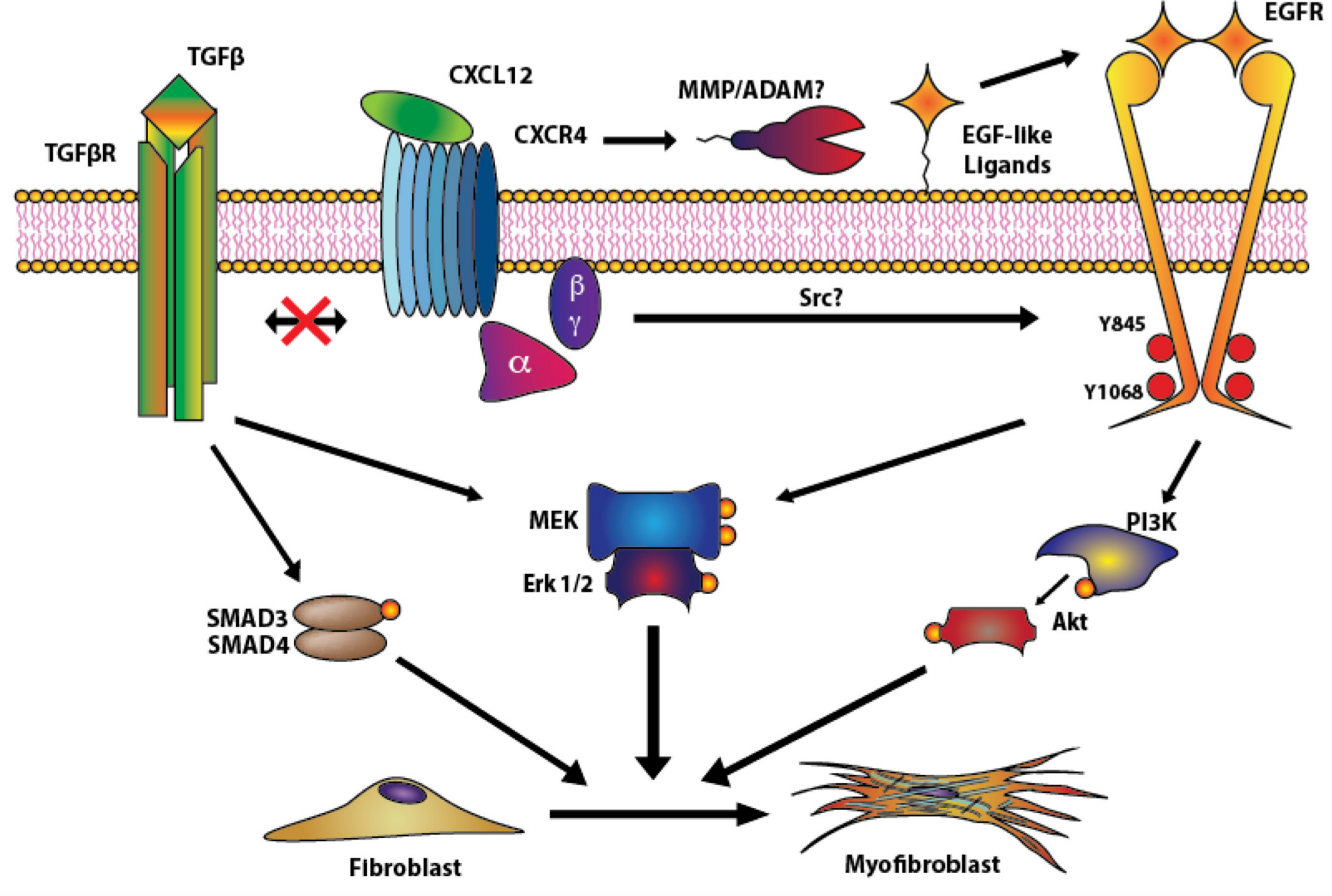
Proposed mechanism of action used by CXCL12 to drive myofibroblast phenoconversion. The canonical pathway driving myofibroblast differentiation is driven by TGF-β-mediated Smad and MEK/Erk signaling^14^. However, upon binding to CXCR4, CXCL12 promotes the transactivation of EGFR, potentially through MMP/ADAM as previously described by Kasina et.al in prostate cancer cells^35^ and Src activation, as shown in the supplementary data (S2 Fig). Active EGFR then activates Akt and MEK/ERK signaling pathways to promote myofibroblast marker gene expression and phenoconversion. CXCL12-mediated phenoconversion acts independently of TGFβR/Smad-mediated myofibroblast phenoconversion.

As shown schematically in Fig 6, our data shows that CXCL12/CXCR4 axis activation promotes the transactivation of the EGFR, potentially through MMP/ADAM protein activation and/or Src activation, and downstream MEK/ERK and PI3K/Akt signaling cascades. The activation of these cascades leads to the expression of myofibroblast markers such as ACTA2 and COL1α1, coincident with the phenoconversion of prostate fibroblasts to myofibroblasts. These events occur in the complete absence of TGFβ and are not coupled to activation of the TGFβ/TGFβR axis. Therefore, the CXCL12/CXCR4 and TGFβ/TGFβR axes function independently to promote prostate myofibroblast phenoconversion.

## Discussion

TGFβ has been heavily documented to influence CXCL12/CXCR4 axis action; specifically TGFβ can modulate the expression and activation of CXCR4 in several systems as well as levels of CXCL12. TGFβ is responsible for increased levels of CXCR4 human hepatic oval cells, T cells, and monkey choroid-retinal endothelial cells[28–30]. TGFβ has also been documented to upregulate CXCL12 in human gingival fibroblasts, tumor-promoting mammary stromal fibroblasts and rat kidney fibroblasts[24,28,31,32]. Interestingly, these relationships between TGFβ and the CXCL12/CXCR4 axis describe a one-way interaction in which TGFβ influences the expression levels and activity of the CXCL12/CXCR4 axis. However, previously our group reported that prostate fibroblasts treated with CXCL12 in serum-free media, which lacks TGFβ in it, would undergo myofibroblast phenoconversion to levels comparable to TGFβ-treated fibroblasts. Therefore, the study presented here focuses on the molecular mechanisms used by CXCL12/CXCR4 to promote myofibroblast phenoconversion in the absence of TGFβ in prostate fibroblasts; since it has been previously documented that CXC-type chemokines are upregulated by aging prostate fibroblasts[33] and these chemokines can achieve myofibroblast differentiation of prostate fibroblast *in vitro* in the absence on TGFβ.

It has been documented previously that EGFR transactivation plays a role in the fibroblast to myofibroblast differentiation of lung fibroblasts through lipid raft-bound CD44 and TGFβ-mediated signaling[34]. However, we observed that EGFR transactivation in prostate fibroblasts by the CXCL12/CXCR4 axis occurred in the absence of TGFβ. We report in this study that activation of the CXCL12/CXCR4 axis leads to a rapid phosphorylation of EGFR and downstream MEK/ERK and PI3K/Akt signaling pathways, and that EGFR transactivation is essential for the expression of myofibroblast markers at the RNA and protein (summarized in Fig 6). Moreover, EGFR transactivation may occur through both extracellular, ADAM/MMP-mediated mechanisms as previously observed in prostate epithelial cells[35], as well as through intracellular phosphorylation of the Y845 residue by Src, which was observed during our experimental procedures (S2 Fig). Thus, CXCL12/CXCR4 axis promotion of myofibroblast phenoconversion is not coupled to canonical TGFβ/Smad mediated signaling.

It was recently reported that TGFβ and CXCL12 work together in an autocrine fashion to promote the differentiation of mammary stromal myofibroblasts[24]. However, our results show no crosstalk between TGFβ and CXCL12 in prostate fibroblasts. Small molecule inhibition of TGFβRI signaling neither augmented, nor ablated, activation of the EGFR or downstream MEK/ERK and PI3K/Akt CXCL12/CXCR4-mediated signaling cascades. More importantly, siRNA-mediated knockdown of CXCR4 and/or TGFβRI had no effect in the activation and downstream gene expression induction by the other receptor by its respective ligand. Knock down or inhibition of TGFβRI had no effect in the CXCL12/CXCR4 axis-mediated expression of myofibroblast markers at the RNA level (Fig 4) and did not inhibit myofibroblast phenoconversion (Fig 6). These results clearly indicate that CXCL12 and TGFβ promote prostate myofibroblast phenoconversion independently, through mechanisms that do not crosstalk or overlap.

Our study provides further insight into prostatic fibrosis, which is a potential pathobiology of benign prostatic disease contributing to lower urinary tract symptoms (LUTS) [8,36]. Other diseases such as idiopathic pulmonary fibrosis (IPF), pancreatic dysfunction and cirrhotic nonalcoholic fatty liver disease include fibrosis as a major component of their pathobiologies[16,37]. Additionally, the data presented in this study further supports the role of EGRF and downstream MEK/ERK activation in the development of tissue fibrosis, as previous studies have correlated the activation of these pathways to IPF development[38,39]. Our group has documented the presence of peri-urethral tissue fibrosis in human[40] and mouse tissues associated with lower urinary tract dysfunction[13], and has shown that myofibroblast phenoconversion can be promoted by pro-inflammatory CXC-type chemokines as well as TGFβ[21]. The data presented in the current study extends these findings and elucidates the signaling mechanisms through which activation of CXCR4 by CXCL12 induces a TGFβ / TGFβR-independent transactivation of EGFR. This transactivation of EGFR is required for downstream signaling by MEK/ERK and PI3K/Akt to induce myofibroblast marker expression and phenoconversion (Fig 6). This transactivation of EGFR occurs in the absence of TGFβ and therefore supports the idea that CXCL12/CXCR4 axis activation may provide an attractive molecular target for novel therapeutics to treat prostatic fibrosis and LUTS.

## Supporting Information

S1 Fig Western blot quantification of Fig 4A and 4B shows that CXCL12-mediated signaling requires CXCR4 and not TGFβRI. Band intensity was quantified using ImageStudio (LiCor), phosphorylated kinases were normalized to total kinase. The fold change was calculated by comparing normalized band intensity to the value of the normalized kinase at 0” for each siRNA.
(TIF)

S2 Fig CXCL12/CXCR4 axis-mediated myofibroblast phenoconversion activates Src. N1 fibroblasts were treated in defined serum-free Ham’s media with CXCL12 (100pM), or 0.01% BSA vehicle. CXCL12 treatment shows Src activation and EGFR-mediated Src activation by the presence of the Y845 phosphorylation at 10 minutes post-treatment.
(TIF)
