# Supplementary figures and images for: CXCL12/CXCR4 Axis Activation Mediates Prostate Myofibroblast Phenoconversion through Non-Canonical EGFR/MEK/ERK Signaling
Source: PLoS One. 2016 Jul 19;11(7):e0159490. doi: 10.1371/journal.pone.0159490 (PMC4951124; doi:10.1371/journal.pone.0159490)

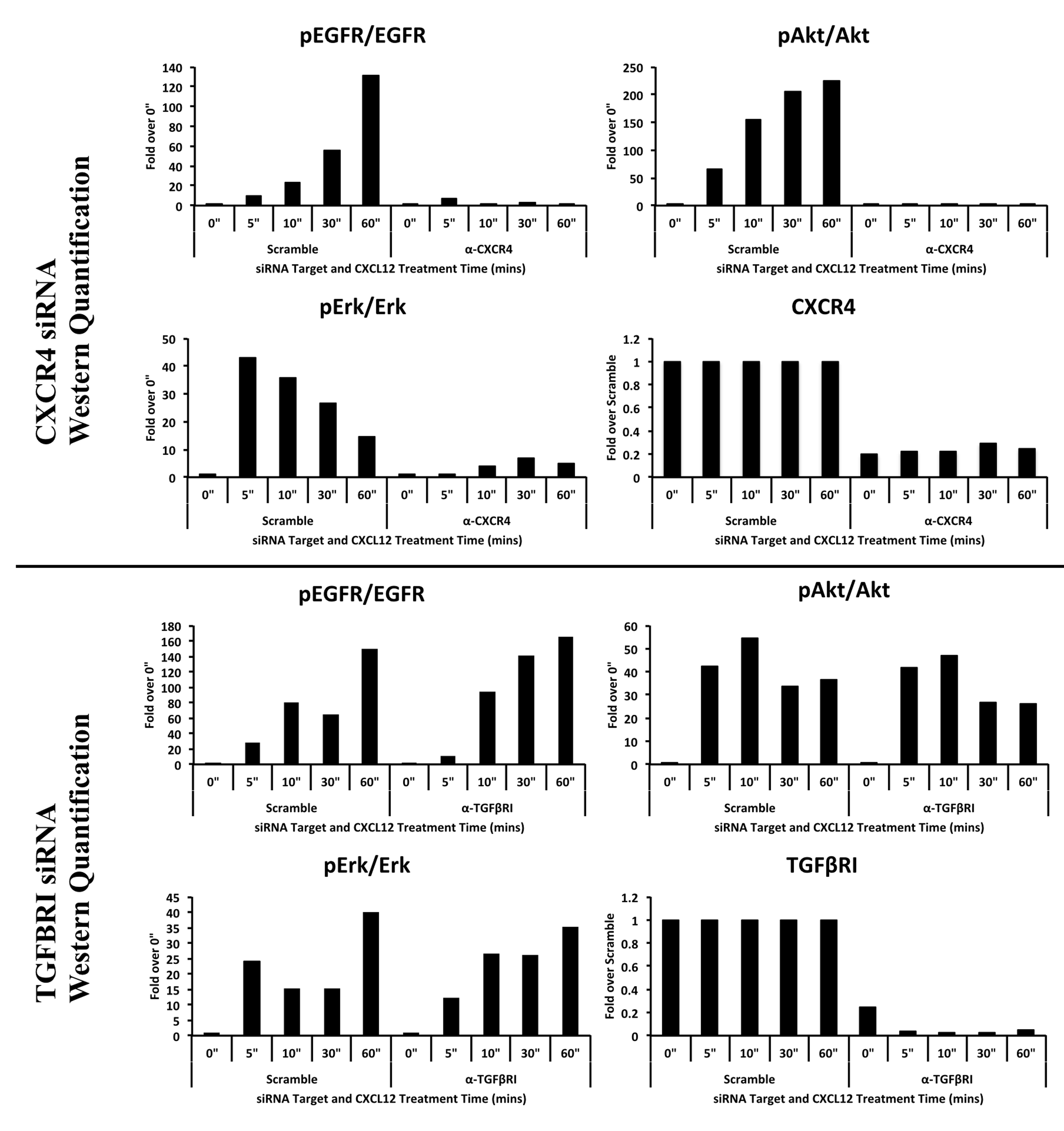

Supplement: S1 Fig — Band intensity was quantified using ImageStudio (LiCor), phosphorylated kinases were normalized to total kinase. The fold change was calculated by comparing normalized band intensity to the value of the normalized kinase at 0” for each siRNA. (TIF) [file pone.0159490.s001.tif]

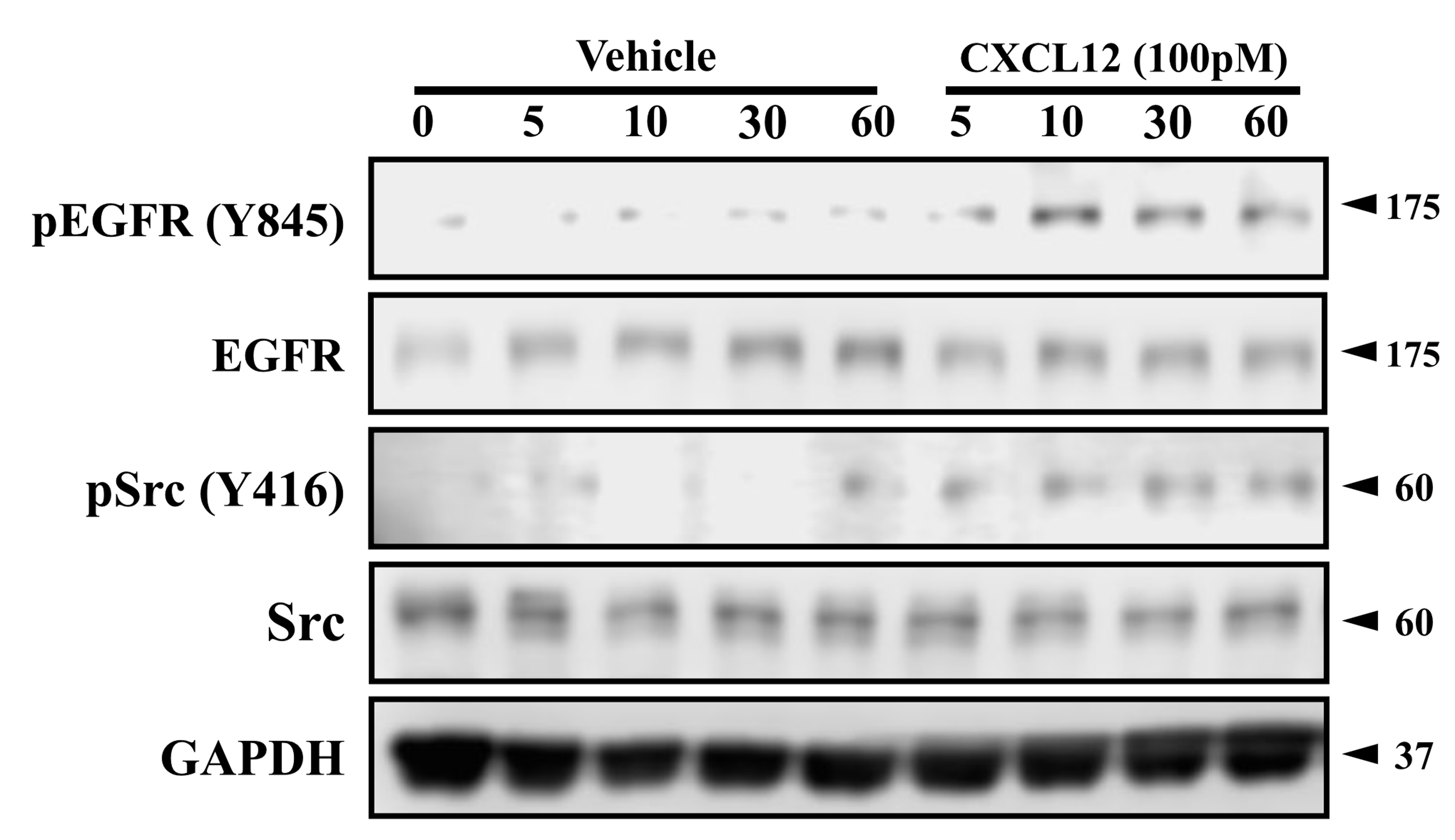

Supplement: S2 Fig — N1 fibroblasts were treated in defined serum-free Ham’s media with CXCL12 (100pM), or 0.01% BSA vehicle. CXCL12 treatment shows Src activation and EGFR-mediated Src activation by the presence of the Y845 phosphorylation at 10 minutes post-treatment. (TIF) [file pone.0159490.s002.tif]
